# Supplementary material for: COZOID: contact zone identifier for visual analysis of protein-protein interactions
Source: BMC Bioinformatics. 2018 Apr 6;19:125. doi: 10.1186/s12859-018-2113-6 (PMC5889581; doi:10.1186/s12859-018-2113-6)
Supplement: Supplementary file 2 — Software build. Executable binary file of the software tool. (ZIP 118,784 kb) [file 12859_2018_2113_MOESM2_ESM.zip › cozoid/java/docs/beansbinding-1.2.1-doc.zip!/org/jdesktop/beansbinding/class-use/PropertyStateListener.html]

Uses of Interface org.jdesktop.beansbinding.PropertyStateListener (Beans Binding)


---


|  |  |  |
| --- | --- | --- |
| **Overview** \| **Package** \| **Class** \| **Use** \| **Tree** \| **Deprecated** \| **Index** \| **Help** | |  |
| PREV   NEXT | **FRAMES**    **NO FRAMES**     **All Classes** |  |


---


## **Uses of Interface org.jdesktop.beansbinding.PropertyStateListener**

| Packages that use PropertyStateListener | |
| --- | --- |
| **org.jdesktop.beansbinding** | Provides support for defining properties and creating bindings between sets of two properties. |

Uses of PropertyStateListener in org.jdesktop.beansbinding

| Methods in org.jdesktop.beansbinding that return PropertyStateListener | |
| --- | --- |
| `PropertyStateListener[]` | `PropertyHelper.getPropertyStateListeners(S source)`             Returns an arry containing the listeners registered for the given source. |
| `abstract  PropertyStateListener[]` | `Property.getPropertyStateListeners(S source)`             Returns an arry containing the listeners registered for the given source. |
| `PropertyStateListener[]` | `ObjectProperty.getPropertyStateListeners(S source)`             Returns an empty array; the state of an `ObjectProperty` never changes so listeners aren't useful. |

| Methods in org.jdesktop.beansbinding with parameters of type PropertyStateListener | |
| --- | --- |
| `void` | `PropertyHelper.addPropertyStateListener(S source, PropertyStateListener listener)`             Adds a `PropertyStateListener` to be notified when the state of the `Property` changes with respect to the given source. |
| `abstract  void` | `Property.addPropertyStateListener(S source, PropertyStateListener listener)`             Adds a `PropertyStateListener` to be notified when the state of the `Property` changes with respect to the given source. |
| `void` | `ObjectProperty.addPropertyStateListener(S source, PropertyStateListener listener)`             Does nothing; the state of an `ObjectProperty` never changes so listeners aren't useful. |
| `void` | `PropertyHelper.removePropertyStateListener(S source, PropertyStateListener listener)`             Removes a `PropertyStateListener` for the given source. |
| `abstract  void` | `Property.removePropertyStateListener(S source, PropertyStateListener listener)`             Removes a `PropertyStateListener` for the given source. |
| `void` | `ObjectProperty.removePropertyStateListener(S source, PropertyStateListener listener)`             Does nothing; the state of an `ObjectProperty` never changes so listeners aren't useful. |

---


|  |  |  |
| --- | --- | --- |
| **Overview** \| **Package** \| **Class** \| **Use** \| **Tree** \| **Deprecated** \| **Index** \| **Help** | |  |
| PREV   NEXT | **FRAMES**    **NO FRAMES**     **All Classes** |  |


---
